# Dispersals of the Siberian Y-chromosome haplogroup Q in Eurasia

**DOI:** 10.1007/s00438-017-1363-8

**Published:** 2017-09-07

**Authors:** Yun-Zhi Huang, Horolma Pamjav, Pavel Flegontov, Vlastimil Stenzl, Shao-Qing Wen, Xin-Zhu Tong, Chuan-Chao Wang, Ling-Xiang Wang, Lan-Hai Wei, Jing-Yi Gao, Li Jin, Hui Li

**Affiliations:** 1 Ministry of Education Key Laboratory of Contemporary Anthropology, School of Life Sciences, Fudan University, Shanghai, 200438 China; 2 National Center of Forensic Experts and Research, Budapest, 1087 Hungary; 3 Department of Biology and Ecology, Faculty of Science, University of Ostrava, 71000 Ostrava, Czech Republic; 4 A.A. Kharkevich Institute for Information Transmission Problems, Russian Academy of Sciences, Moscow, 127051 Russian Federation; 5 Institute of Criminalistics, Police of the Czech Republic, 17089 Prague, Czech Republic; 6 Department of Anthropology and Ethnology, Xiamen University, Xiamen, 361005 China; 7 Institut National des Langues et Civilisations Orientales, 75013 Paris, France; 8 Faculty of Arts and Humanities, University of Tartu, 50090 Tartu, Estonia; 9 Faculty of Central European Studies, Beijing International Studies University, Beijing, 100024 China

**Keywords:** Y-chromosome, Haplogroup Q, Multidirectional migrations, Eurasia, Han Chinese

## Abstract

The human Y-chromosome has proven to be a powerful tool for tracing the paternal history of human populations and genealogical ancestors. The human Y-chromosome haplogroup Q is the most frequent haplogroup in the Americas. Previous studies have traced the origin of haplogroup Q to the region around Central Asia and Southern Siberia. Although the diversity of haplogroup Q in the Americas has been studied in detail, investigations on the diffusion of haplogroup Q in Eurasia and Africa are still limited. In this study, we collected 39 samples from China and Russia, investigated 432 samples from previous studies of haplogroup Q, and analyzed the single nucleotide polymorphism (SNP) subclades Q1a1a1-M120, Q1a2a1-L54, Q1a1b-M25, Q1a2-M346, Q1a2a1a2-L804, Q1a2b2-F1161, Q1b1a-M378, and Q1b1a1-L245. Through NETWORK and BATWING analyses, we found that the subclades of haplogroup Q continued to disperse from Central Asia and Southern Siberia during the past 10,000 years. Apart from its migration through the Beringia to the Americas, haplogroup Q also moved from Asia to the south and to the west during the Neolithic period, and subsequently to the whole of Eurasia and part of Africa.

**Electronic supplementary material:**

The online version of this article (doi:10.1007/s00438-017-1363-8) contains supplementary material, which is available to authorized users.

## Introduction

In recent decades, the human Y-chromosome has proven to be a powerful tool for tracing the paternal history of human populations and genealogical ancestors. The human Y-chromosome haplogroup Q (also named Q-M242 in accordance with its defining mutation) probably originated in Central Asia and Southern Siberia during the time period of 15–25 KYA (1000 years ago) (Karafet et al. 2002, 2008; Bortolini et al. 2003; Seielstad et al. 2003), then subsequently diffused in the eastward, westward and southward directions (Zhong et al. 2011; Di Cristofaro et al. 2013; Sandoval et al. 2013; Liu et al. 2014; Rasmussen et al. 2014). Haplogroup Q has several subclades defined by single nucleotide polymorphisms (SNPs), and it reaches its highest frequency of 70–100% in the Americas (Bortolini et al. 2003; Seielstad et al. 2003; Zhong et al. 2011; Rasmussen et al. 2014). Although the diversity of haplogroup Q in the Americas has been studied in detail (Bisso-Machado et al. 2011; Toscanini et al. 2011, 2016; Jota et al. 2011; Malyarchuk et al. 2011; Dulik et al. 2012b; Battaglia et al. 2013; Lardone et al. 2013; Melton et al. 2013; Regueiro et al. 2013; Noguera et al. 2014; Sala and Corach 2014; Torres et al. 2015), investigations on the diffusion of haplogroup Q in Eurasia and Africa are still limited. Consequently, we studied samples of haplogroup Q in Eurasia to explore how it expanded from Central Asia and Southern Siberia during the Neolithic period.

The ancestors of present-day Native Americans migrated to the Americas from Siberia via the Beringia around 16 KYA (Raghavan et al. 2015; Llamas et al. 2016). Q1a2a1-L54 and its subclade Q1a2a1a1-M3 are the two predominant subclades of haplogroup Q found on both sides of the Bering Strait. Q1a2a1-L54 has spread throughout Northern Asia, the Americas, and Western and Central Europe (Raff and Bolnick 2014; Rasmussen et al. 2014). An ancient individual of the Clovis culture belonged to Q1a2a1-L54 (xQ1a2a1a1-M3) (O’Rourke and Raff 2010; Rasmussen et al. 2014). Q1a2a1a1-M3, one of the most thoroughly studied subclades within haplogroup Q, is frequent both in the Chukotka Peninsula of Siberia (close to Alaska) and the Americas (Lell et al. 2002). Previous studies indicated that Q1a2a1a1-M3 migrated from Siberia to the Americas and partially returned to Siberia (Hammer et al. 1997; Lell et al. 1997; Bortolini et al. 2003; Pakendorf et al. 2007). The estimated time of Q1a2a1a1-M3 is 13-22 KYA (Dulik et al. 2012a). Q1a2a1a1a-M19, a subclade of Q1a2a1a1-M3, remained in Southern America and has a similarly diversified pattern with its upstream lineage. The age of Q1a2a1a1a-M19 is approximately 7–8 KYA (Bortolini et al. 2003; Jota et al. 2011).

The frequencies of haplogroup Q range from 0 to 94% in Eurasia (approximately 5% on average) (Seielstad et al. 2003; Varzari et al. 2013). Haplogroup Q reaches its highest frequencies in Siberia, especially in Kets (90–94%) and Selkups (66–71%), and is rarely seen in Western, Southern and South-eastern Asia (Wells et al. 2001; Zerjal et al. 2002; Tambets et al. 2004; Sengupta et al. 2006; Sharma et al. 2007; Haber et al. 2011a, b; Dulik et al. 2011; Grugni et al. 2012; Di Cristofaro et al. 2013; Rasmussen et al. 2014). Subclade Q1a1a1-M120 appears almost only in Eastern Asia, and its diversity implies that haplogroup Q has migrated from north to south with the ancestors of current Han Chinese during the Neolithic period (Su et al. 2000; Wells et al. 2001; Tarazona-Santos et al. 2001; Wen et al. 2004; Gayden et al. 2007; Nonaka et al. 2007; Zhong et al. 2011; Zhao et al. 2015). Subclades Q1a1b-M25 and Q1a2-M346 have spread widely in Eurasia. Q1a1b-M25 reaches its highest frequency in Turkmen (34–43%) and shows low frequencies in other Eurasian populations (Underhill et al. 2000; Malyarchuk et al. 2011; Zhong et al. 2011), while Q1a2-M346 appears in Central, Western and Southern Asia, and most parts of Europe (Sengupta et al. 2006; Sharma et al. 2007; Abu-Amero et al. 2009; Bailliet et al. 2009).

Haplogroup Q has also appeared in other parts of the world. For instance, an ancient DNA study of a Saqqaq individual in Greenland suggests that haplogroup Q1a-MEH2 was frequent in Siberian and Native American populations (Karafet et al. 2008; Rasmussen et al. 2010; Raghavan et al. 2015). A few subclades of haplogroup Q have been identified in the Comoros population in Africa (Q1a2-M346) and the Polynesian islands in Oceania (Q1a2a1a1c-M199) (Hurles et al. 2003; Msaidie et al. 2010).

Nowadays, the distribution of haplogroup Q in the Americas has been studied thoroughly, but we know little about its dispersals on western and southern routes. In this study, we present an analysis of some SNP subclades of haplogroup Q, including Q1a1a1-M120, Q1a2a1-L54, Q1a1b-M25, Q1a2-M346, Q1a2a1a2-L804, Q1a2b2-F1161, Q1b1a-M378, and Q1b1a1-L245. Based on NETWORK and BATWING analyses of haplogroup Q, we were able to better understand its dispersals on western and southern routes, and their impacts on Eurasian populations.

## Materials and methods

### Ethic statement

This study was conducted after the approval of the Ethical Committee of the School of Life Sciences, Fudan University (Shanghai, China) and the ethical committee of the Lomonosov Moscow State University (Moscow, Russia). All donors of samples were completely informed and signed informed consent forms before sample collection.

### Population samples

In this study, a total of 471 unrelated male samples were analyzed. We collected blood samples of 1757 healthy and unrelated volunteers from five populations in China, including 700 Hui, 64 Bao-An, 109 Dong-Xiang, 90 Li-Qian, and 794 Shao-Xing individuals. In addition, we collected saliva samples of 30 healthy and unrelated volunteers from 3 populations in Russia, including 4 Enets, 19 Ket, and 7 Selkup individuals. After genotyping all samples, we confirmed that 16 samples of China and 23 samples of Russia belonged to haplogroup Q, which were further investigated in this study. Furthermore, data from previous studies were also analyzed (Bailliet et al. 2009; Zhong et al. 2011; Lacau et al. 2012; Dulik et al. 2012; Di Cristofaro et al. 2013; Sandoval et al. 2013; Varzari et al. 2013; Hollard et al. 2014; Liu et al. 2014; Family Tree DNA). The populations were categorized in accordance with the location of residence as follows: from Gansu province of China: Bao-An, one individual from Ji-Shi Mountain; Dong-Xiang, two individuals from Dong-Xiang county, Hui Autonomous Prefecture of Lin-Xia; Li-Qian, four individuals from Yong-Chang county, Jin-Chang city; from Zhejiang Province of China: Shao-Xing, nine individuals from Shao-Xing city. In the Krasnoyarsk Region of Russia: Enets—two individuals from Potapovo; Ket—one individual from each of Farkovo, Sulomai/Bor, Sumarokovo, Turukhansk, and Verkhneimbatsk, two individuals from each of Bakhta, Baklanikha and Kellog, and five individuals from Sulomai; Selkup—three individuals from Farkovo, and two individuals from Turukhansk. These three populations are considered minorities in Russia according to the 2002 All-Russia Population Census (ESM_3). Enets (named Entses in ESM_3) has 237 individuals; Ket has 1494 individuals; Selkup has 4249 individuals.

### Y-chromosome markers

Genomic DNA was extracted from the blood samples using the DP-318 Kit (Tiangen Biotechnology, Beijing, China), and the DNA extraction protocol for the saliva samples was adapted from the high-salt DNA extraction method (Quinque et al. 2006). The samples were typed as the most recent Y-chromosome phylogenetic tree (ISOGG 2017). The selected samples belonged to several subclades of haplogroup Q.

Binary markers were hierarchically genotyped by SNaPshot (ABI SNaPshot Multiplex Kit, Carlsbad, CA, USA) and fluorescent allele-specific PCR. The PCR products were electrophoresed on a 3730xl Genetic Analyzer (Applied Biosystems, Carlsbad, CA, USA). Seventeen Y-chromosomal STRs (DYS19, DYS389I, DYS389II, DYS390, DYS391, DYS392, DYS393, DYS385a, DYS385b, DYS438, DYS439, DYS437, DYS448, DYS456, DYS458, DYS635 and YGATAH4) were amplified using the AmpFlSTR Yfiler PCR amplification kit (Applied Biosystems). The amplified products were separated and identified using a 3730xl Genetic Analyzer (Applied Biosystems) according to the protocol recommended by the manufacturer. The data were analyzed using a Gene-Mapper ID v3.2 (Applied Biosystems). In the analyses, DYS389II was calculated by subtracting the DYS389I allele size.

### Statistical analyses

Networks of Y-chromosomal STR data were constructed by the reduced-median method using NETWORK v. 5.0.0.1 (http://www.fluxus-engineering.com) with haplogroups Q1a1a1-M120, Q1a2a1-L54, Q1a1b-M25, Q1a2-M346, Q1a2a1a2-L804, Q1a2b2-F1161, Q1b1a-M378, and Q1b1a1-L245. Because we collected samples from different studies, we had to adjust the number of loci used in our study to match those of other studies. The network of Q1a1a1-M120 was constructed with seven loci: DYS19, DYS389I, DYS389II, DYS390, DYS391, DYS392, and DYS393. The network of Q1a2a1-L54 was constructed with 15 loci: DYS19, DYS389I, DYS389II, DYS390, DYS391, DYS392, DYS393, DYS437, DYS438, DYS439, DYS448, DYS456, DYS458, DYS635 and GATA H4. The networks of Q1a1b-M25, Q1a2-M346, Q1a2a1a2-L804, Q1a2b2-F1161, Q1b1a-M378, and Q1b1a1-L245 were each constructed with ten loci: DYS19, DYS389I, DYS389II, DYS390, DYS391, DYS392, DYS393, DYS437, DYS438 and DYS439.

We used the Markov chain Monte Carlo (MCMC) approach (Wilson et al. 2003) incorporated into the program BATWING to estimate the time to the most recent common ancestor (TMRCA) and the expansion time of the aforementioned Q subclades. Time estimates for subclades of haplogroup Q were made using seven to fifteen of the STRs listed above. A model of exponential growth from an initially constant-sized population was employed in BATWING for obtaining the time estimates. Four sets of widely used Y-STR mutation rates were applied in the time estimates as Wei et al. (2013): evolutionary mutation rate (EMR) (Zhivotovsky et al. 2004), two observed genealogical mutation rates (OMRB and OMRS) (Shi et al. 2010; Burgarella and Navascués 2011), and a genealogical mutation rate adjusted for population variation using a logistic model (lmMR) (Wilson et al. 2003). A generation time of 30 years was used to produce a time estimate in years (Tremblay and Vézina 2000). We applied weakly informative prior distribution parameters in BATWING estimations to analyze populations individually. For the initial effective population size (*N*), we used a broad prior gamma (1, 0.0001) (mean = 10,000, SD = 10,000). For population growth rate per generation (*α*), we also used the broad prior distribution gamma (2, 400) (mean = 0.005, SD = 0.0035). For the time in coalescent units when exponential growth (*β*) began we used gamma (2, 1) (mean = 2, SD = 1.41) (Xue et al. 2006). A total of 10^4^ samples of the program’s output representing 10^6^ MCMC cycles were taken after discarding the first 3 × 10^3^ samples as “burn-in” (Xue et al. 2006), and convergence was confirmed by examining longer runs for all populations and finding the same posterior distributions. The TMRCA was calculated using the product of the estimated population size *N* and the height of the tree *T* (in coalescent units).

**Fig. 1:**
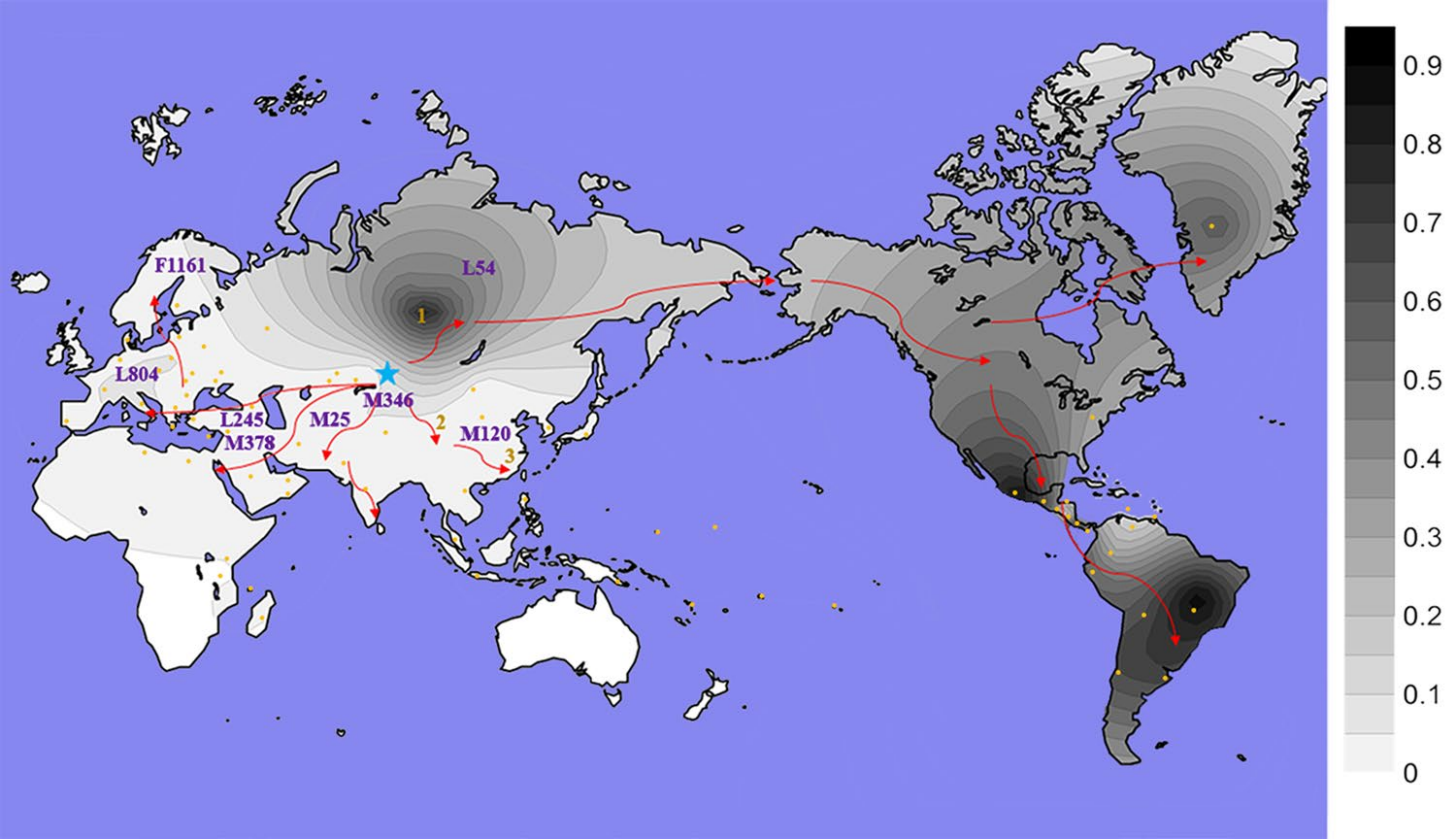
Worldwide distribution of haplogroup Q-M242. The *blue star* is the original place of haplogroup Q-M242, around Central Asia and Siberia. The *brown number one* is Russian sample location in the Krasnoyarsk Region. The *brown number two* is Chinese sample location in Gansu province. The *brown number three* is Chinese sample location in Zhejiang province. The *red arrows* are the expansion routes of haplogroup Q-M242. The *purple words* show the locations of subclades of haplogroup Q used in this study. The *orange points* represent the sample locations collected from published studies (ESM_2) (color figure online)

A contour map for the frequencies of haplogroups Q-M242 was generated using the Kriging procedure with the aid of the Golden Software Surfer 11 (Golden Software Inc., CO, USA) (Fig. 1). Since the frequency data were obtained from many sources, the identified subclades of haplogroup Q were different. To show all frequencies in one figure, we integrated the frequencies of different subclades into frequencies of Q-M242. The raw frequency data and references are shown in ESM_2.

## Results

### Worldwide distribution of haplogroup Q-M242

**Fig. 2:**
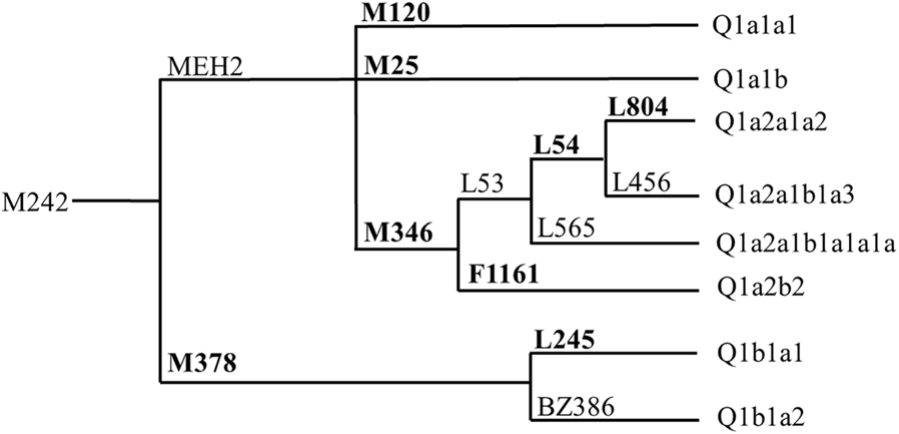
Phylogenetic tree of Y-chromosome haplogroup Q-M242. The haplogroup labeling is in agreement with the ISOGG conventions and recent updates (ISOGG 2017). The used subclades are showed in *bold*

We calculated the frequencies of our samples and collected the frequency data from previous studies (ESM_2). As can be seen in Fig. 1, the frequencies of haplogroup Q-M242 are low in most of the world, except for the Americas and a small part of Siberia, which matches previously published observations on the distribution of haplogroup Q (Balanovsky et al. 2017). Moreover, we represented the migration routes of haplogroup Q-M242 based on our results and previous studies (Fig. 1, ESM_2). We also marked the main distribution regions of the subclades studied in this research (Fig. 1). We have constructed a phylogenetic tree within haplogroup Q to easily identify the downstream subclades (Fig. 2).

### The network of haplogroup Q subclades

**Fig. 3:**
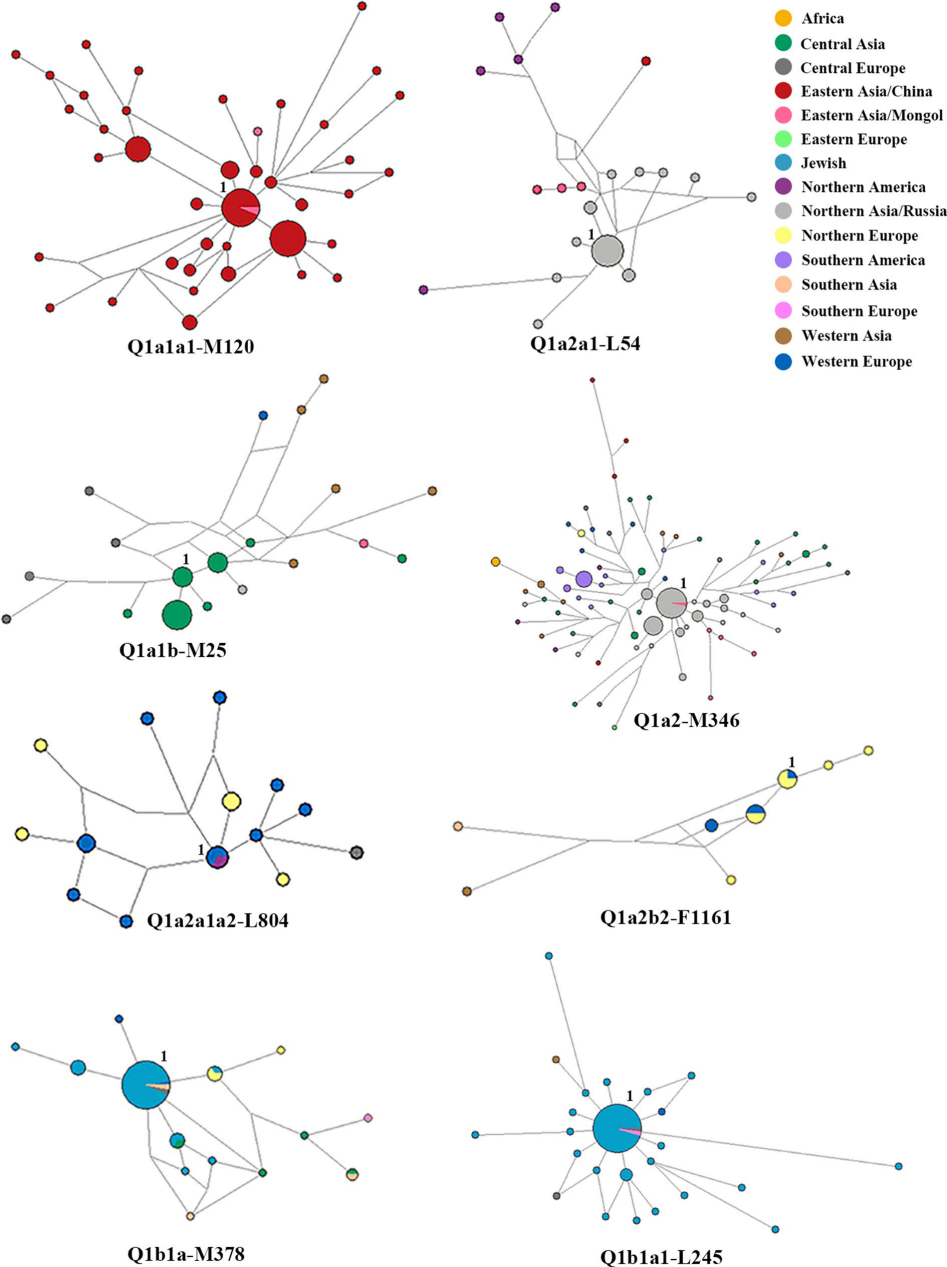
Network of Y-STR haplotypes within haplogroup Q-M242. Q1a1a1-M120: cluster 1 is shared by 16 Eastern Asian/China samples and one Eastern Asian/Mongolia. Q1a2a1-L54: cluster 1 is shared by 11 Northern Asian/Russian samples. Q1a1b-M25: cluster 1 is shared by five Central Asian samples. Q1a2-M346: cluster 1 is shared by 36 Northern Asian samples and 1 Eastern Asian/Mongolia. Q1a2a1a2-L804: cluster 1 is shared by two Western European samples and one Northern American sample. Q1a2b2-F1161: cluster 1 is shared by three Northern European samples and one Western European sample. Q1b1a-M378: cluster 1 is shared by 29 Jewish samples (14 from Central Europe; eleven from Eastern Europe; three from Southern Europe; one from Western Asia), one Western European sample, one Central European sample and one Southern Asian samples. Q1b1a1-L245: cluster 1 is shared by 37 Jewish samples (16 from Central Europe; 14 from Eastern Europe; four from Western Asia; two from Southern Europe; one from Western Europe), one Western European sample and one Southern European sample. Samples included in every cluster 1 are colored by *purple* in ESM_1

To reveal the detailed structures for subclades of haplogroup Q, we conducted a network analysis combining the SNP and the STR haplotype data for 471 individuals (Fig. 3). The network of Q1a1a1-M120 included most samples from China along with a small number of Mongolian samples. The network of Q1a2a1-L54 contained most samples from Siberia (Northern Asia/Russia), and few samples from Mongolia, China and Northern America. The network of Q1a1b-M25 consisted of samples from Central Asia with a small number of Eastern Asian/Mongolian, Western Asian, Central and Western European samples. The network of Q1a2-M346 mostly contained samples from Northern Asia/Russia and Mongolia, and a few samples from Asia, Europe, the Americas, and even Africa. The network of Q1a2a1a2-L804 had a central cluster shared by Western European and Northern American samples with other branches of Western, Northern and Central Europe. The network of Q1a2b2-F1161 was mainly composed of samples from Northern and Western Europe with two single branches of Southern and Western Asia. The network of Q1b1a-M378 was mainly composed of Jewish samples and a small number of Southern and Central Asian, Western, Northern and Southern European samples. The network of Q1b1a1-L245 had a star-like shape of Jewish samples and a small amount of European and Western Asian samples. We did not discuss the origins and migrations of samples from the Americas because we focused on the dispersals of haplogroup Q in Eurasia and just used samples from the Americas to construct the network.

### Time estimates for haplogroup Q

We used BATWING to estimate the TMRCA and the expansion time for the subclades of haplogroup Q. As seen in Table 1, the three genealogical mutation rates had approximately similar results, while using the evolutionary mutation rate resulted in a much older TMRCA. The genealogical mutation rates were more reliable when we analyzed a large number of loci and closely related individuals, whereas the evolutionary mutation rate tended to be more effective for estimates on a smaller number of loci and genetically distant individuals (Wang et al. 2014; Wang and Li 2015). Since we used from seven to fifteen loci in the time estimates, and the used populations belonged to the same subclades of haplogroup Q, we decided to use the results of the three genealogical mutation rates.

**Table 1:** The TMRCA and expansion times of haplogroup Q subclades (KYA)

Branch (region or population)	EMR (TMRCA/expansion time)	OMRB (TMRCA/expansion time)	OMRS (TMRCA/expansion time)	lmMR (TMRCA/expansion time)
Q1a1a1-M120	38.5/16.2	11.1/6.4	11.1/6.4	9.8/5.8
Q1a1a1-M120 DYS391 allele 6 (Han Chinese)	20.7/13.0	6.5/5.5	6.7/5.6	5.6/5.0
Q1a1a1-M120 DYS391 allele 9 (Han Chinese)	24.2/13.7	7.6/5.8	7.8/5.9	6.5/5.2
Q1a2a1-L54 (Yenisei basin)	10.4/9.2	2.5/3.4	2.4/3.3	0.3/0.4
Q1a1b-M25 (Turkic)	10.9/5.3	3.3/2.2	4.6/2.7	3.1/2.0
Q1a2-M346 (Turkic)	13.4/8.0	4.5/3.8	5.5/4.4	4.0/3.6
Q1a2-M346 (Western Asia)	15.1/11.4	4.4/4.6	5.8/5.5	4.0/4.3
Q1a2-M346 (Europe)	14.0/10.0	4.1/4.1	5.2/4.9	3.8/3.9
Q1a2a1a2-L804	19.0/12.6	5.8/5.5	7.1/6.3	5.3/5.2
Q1a2b2-F1161	8.6/6.4	2.6/2.6	3.3/3.1	2.3/2.4
Q1b1a-M378 (Jews)	4.9/4.0	1.5/1.6	1.8/1.8	1.4/1.5
Q1b1a-M378 (Europe)	6.0/5.2	1.8/2.1	2.4/2.6	1.7/1.9
Q1b1a-M378 (Southern Asia)	4.0/7.0	1.1/3.5	1.5/4.1	1.0/3.4
Q1b1a1-L245 (Jews)	23.2/9.1	9.8/5.0	10.9/5.4	8.6/4.8
Q1b1a1-L245 (Western Asia)	14.6/11.4	4.1/4.5	5.6/5.5	3.9/4.3
Q1b1a1-L245 (Southern Asia)	7.1/6.1	2.1/2.5	2.7/2.9	1.9/2.3

*TMRCA* the time to the most recent common ancestor, *KYA* thousand years ago, *EMR* evolutionary mutation rate (EMR) (Zhivotovsky et al. 2004), *OMRB* observed genealogical mutation rate (Shi et al. 2010), *OMRS* observed genealogical mutation rate (Burgarella and Navascués 2011), *lmMR* a genealogical mutation rate adjusted for population variation using logistic model (Wilson et al. 2003)

## Discussion

Subclade Q1a1a1-M120 was found specifically in the Han Chinese with a low frequency (Zhong et al. 2011). Our results suggested that subclade Q1a1a1-M120 had migrated from Mongolia to China during the Neolithic period, and spread over China with the ancestors of Han Chinese (Fig. 3; Table 1; ESM_1). Previous studies showed that Q1a1a1-M120 had migrated from north-western China to the Central Plain as nomads, and merged into the northern Han Chinese farmers at approximately 2.5–3 KYA (Zhao et al. 2010, 2014, 2015; Yan et al. 2014). Therefore, we supposed that the ancient nomads with Q1a1a1-M120 had migrated to south-eastward from north-western China and were assimilated by the Han Chinese farmers (Zhao et al. 2015).

Subclade Q1a2a1-L54 was mainly found in Yeniseian (Ket) and Samoyedic (Enets and Selkup) speakers (ESM_1). Genetic evidence showed that Yeniseian and Samoyedic speakers had genetic affinities to northern Altaians with high frequencies of haplogroup Q-M242 (xL54), while southern Altaians had many L54 samples and showed similarities with Turkic-speaking populations (Dulik et al. 2012b; Battaglia et al. 2013; Flegontov et al. 2016). However, Yeniseian and Samoyedic samples in this study belonged to L54, which was different from the results of previous studies (xL54). In view of the time estimates (Table 1), we postulated that Q1a2a1-L54 had migrated from the southern Altai region and was assimilated into Yeniseian- and Samoyedic-speaking populations during a recent historical period.

Both Q1a1b-M25 and Q1a2-M346 subclades were frequent in Turkic-speaking populations, and their time estimates were at approximately 3-5 KYA (ESM_1; Table 1). According to Fig. 3 and Table 1, Q1a1b-M25 had spread from Central Asia to Western Asia and to Hungary in Central Europe (ESM_1); Q1a2-M346 had migrated from Southern Siberia (Malyarchuk et al. 2011) to most parts of Eurasia and the Comoros Islands of Africa. The results coincided with Turkic nomadic migrations from Southern Siberia and Mongolia to Central and Western Asia, Caucasus, and Eastern Europe (Yunusbayev et al. 2015). Therefore, we suggested that Q1a1b-M25 and Q1a2-M346 probably migrated with Turkic nomads from Southern Siberia to most parts of Eurasia. A few Q1a1b-M25 and Q1a2-M346 samples in Mongolic-speaking populations probably indicated that Turkic nomads had overlapped with Mongolic-speaking populations when they lived in the present Mongolian territory (Yunusbayev et al. 2015). An ancient DNA study showed that the Hungarians probably originated from Central Asia–Southern Siberia at approximately 4 KYA (Neparáczki et al. 2016), which was consistent with our time estimates (Table 1). Therefore, we proposed that Q1a1b-M25 and Q1a2-M346 had migrated from Central Asia–Southern Siberia to Central Europe at least 4 KYA. Three individuals of Africa (the Comoros Islands) that belonged to Q1a2-M346 reaffirmed that Middle Eastern populations had a genetic influence on the Comoros Islands (Gourjon et al. 2011).

Subclades Q1a2a1a2-L804 and Q1a2b2-F1161 were the downstream of Q1a2-M346 (Fig. 2), both of which mainly distributed in Western and Northern Europe (Fig. 3). Q1a2a1a2-L804 arrived in Western and Northern Europe as early as 5-7 KYA (Table 1). Ancient DNA studies showed that first European farmers migrated from Central Europe to Western and Northern Europe between 5 and 7.5 KYA (Haak et al. 2005, 2010; Bramanti et al. 2009; Malmström et al. 2009). Therefore, we supposed that Q1a2a1a2-L804 had spread from Central Europe to Western and Northern Europe with European early Neolithic farmers. The time estimate for Q1a2b2-F1161 was one thousand years later than its upstream clade Q1a2-M346 (Table 1), which seemed to be unrelated to the Neolithic transition of Europe (Haak et al. 2010). Since Q1a2-M346 spread across Europe at that time, it probably brought Q1a2b2-F1161 to Western and Northern Europe, and even to Western and Southern Asia (Khurana et al. 2014; Yunusbayev et al. 2015).

Subclades Q1b1a-M378 and Q1b1a1-L245 were correlated with the Jewish people, both of which probably represented that some of the Jewish Diaspora populations had expanded into Europe within historical times (Table 1; Fig. 3). As seen in Fig. 3, the central clusters of Q1b1a-M378 and Q1b1a1-L245 mainly consisted of samples from Central and Eastern Europe. The results reaffirmed that some Jewish Diaspora populations had migrated from Central and Eastern Europe, and finally settled in other parts of Europe (Nogueiro et al. 2010; Zoossmann-Diskin 2010). Previous Y-chromosome studies showed that haplogroups J, R and Q3a1 had certain proportions in Jewish populations and spread over Europe (Nogueiro et al. 2010; Chaubey et al. 2016; Balanovsky et al. 2017). Subclades Q1b1a-M378 and Q1b1a1-L245 probably spread over Europe with haplogroups J, R and Q3a1. The Q1b1a-M378 samples from Southern Asia might represent the descendants of Ashkenazi Jewish populations because its upstream haplogroup Q-P36 was regarded as minor Ashkenazi Jewish founding lineages in Southern Asia (Lee et al. 2014).

Our study of the human Y-chromosome haplogroup Q in Eurasia revealed a clear pattern of its migration routes during the past 10,000 years, especially in Han Chinese, Yeniseian-, Samoyedic-, Turkic- speaking and Jewish populations. It is clear that a higher resolution database will be helpful to draw more conclusions on the origins, migrations, and ethno-linguistic affiliations of haplogroup Q.

## Electronic supplementary material

Below is the link to the electronic supplementary material.

Y-chromosomal STR data haplotypes used within haplogroup Q (XLSX 59 kb)

Frequencies of Haplogroup Q in the world (XLSX 47 kb)

National composition of population and citizenship in Russia (XLSX 25 kb)
